# Effects of *Wx* and Its Interaction With *SSIII-2* on Rice Eating and Cooking Qualities

**DOI:** 10.3389/fpls.2018.00456

**Published:** 2018-04-10

**Authors:** Bowen Yang, Shunju Xu, Liang Xu, Hui You, Xunchao Xiang

**Affiliations:** ^1^Laboratory of Plant Molecular Genetics and Breeding, Southwest University of Science and Technology, Mianyang, China; ^2^Engineering Research Center for Biomass Resource Utilization and Modification of Sichuan Province, Mianyang, China

**Keywords:** rice (*Oryza stativa* L.), *Wx*, *SSIII-2*, single nucleotide polymorphism, eating and cooking qualities, epistasis

## Abstract

The *Wx* gene encodes a granule-bound starch synthase (GBSS) and plays a key role in determining rice eating and cooking qualities (ECQs). SSIII-2 (SSIIIa), a member of the soluble starch synthases, is responsible for the synthesis of long chains of amylopectin. To investigate the effects of *Wx* and its interaction with *SSIII-2* on grain ECQs, a population from a hybrid combination was established as a research material. The genotypes of *SSIII-2* and the single nucleotide polymorphisms (SNPs) on intron1, exon6, and exon10 of *Wx*, and the physicochemical indicators and rapid visco analyzer (RVA) profile characteristics were analyzed. The results revealed various effects of *SSIII-2* on rice quality under different backgrounds of *Wx* alleles. There was no obvious difference between different *SSIII-2* alleles under the same background of *Wx*^*a*^, whereas there was a significant diversity under the same background of *Wx*^*b*^. *Wx*^*a*^ had a dominant epistasis to *SSIII-2* because the effect of *SSIII-2* was masked by the massive synthesis of GBSS under *Wx*^*a*^. The apparent amylose content (AAC) was mainly controlled by the In1G/T SNP, and rice gel consistency (GC) was regulated by the Ex10C/T SNP. The combined effects of three SNPs had a significant influence on all ECQs and RVA profile parameters, except for gelatinization temperature. In1T-Ex6A-Ex10C and In1T-Ex6A-Ex10T were classified as being low AAC type. TT-AA-CC and TT-AA-TT had a low AAC and a soft GC. The combined effects of different SNPs of *Wx* are very important for rice quality breeding.

## Introduction

Rice yield has increased significantly over the past decade due to the introduction of semi-dwarfness and the exploitation of heterosis. However, with the constantly increasing demands from society, rice quality cannot meet the requirements of consumers. Despite many studies that have attempted to improve rice quality (Aluko et al., 2004; Zhou et al., 2010; Nelson et al., 2011), the genetic mechanism that controls quality remains ambiguous, and only a few quality-related genes have been identified (Li et al., 2011, 2014; Wang et al., 2012).

Rice quality is determined by starch, which is composed of two polysaccharides: amylose and amylopectin. High amylose content (AC) varieties have firm and separate grains once cooked, whereas low AC cultivars are tender, glossy, and cohesive (Jobling, 2004). The percentage of amylose content in total starch is measured as the apparent amylose content (AAC) using a spectrophotometer, because it contains both of amylose and long-chain amylopectin content. According to the standard Chinese rice resources evaluation, AAC can be classified as waxy (0–2%), very low (2–10%), low (10–20%), intermediate (20–25%), and high (25–33%). In addition, the most important indices of grain quality are eating and cooking qualities (ECQs) that are determined by three physical and chemical indices: AAC, rice gel consistency (GC), and gelatinization temperature (GT). A rice starch rapid visco analyzer (RVA) is widely used to evaluate rice grain quality because it provides a rapid and accurate determination of ECQs for a small amount of sample (Bao and Xia, 1999).

The *Wx* gene, encoding a granule-bound starch synthase (GBSS), was reported to be the main controller of AAC (Nakamura, 2002; Septiningsih et al., 2003; Li et al., 2010). Moreover, *Wx* also plays a key role in determining the physicochemical properties, such as GC, GT, and most RVA parameters (Tan et al., 1999; Fan et al., 2005; He et al., 2006; Wang et al., 2007). The Waxy gene is located on chromosome 6 and consists of 13 exons and 12 introns. Various single nucleotide polymorphisms (SNPs) of *Wx* were found, including a G to T (G-T) SNP of the first intron, A to G (A-G) SNP of the fourth exon, A to C (A-C) SNP of the sixth exon, and C to T (C-T) SNP of the tenth exon (Sano, 1984; Mikami et al., 2008). At least four alleles of *Wx* (*Wx*^*a*^*, Wx*^*b*^*, Wx*^*op*^, and *Wx*^*in*^) have been identified by these nucleotide polymorphisms (Wanchana et al., 2003). The *wx* mutant allele was caused by a 23-bp insertion in its second exon, which led to a premature expression of Wx protein. Mutations of *Wx* are associated with the ECQs of rice. For example, the G-T single nucleotide variation of the intron 1 has a strong influence on the normal expression of *Wx*. All rice varieties with a G express *GBSS* mRNA at normal levels. In contrast, the transcript level of *GBSS* in rice varieties with a T was lower (Wang et al., 1995). Ayres et al. (1997) found that this difference in the expression of *GBSS* was caused by a single-base change (G-T) in the putative 5′ leader intron splice site that interfered with mRNA processing. The allele *Wx*^*in*^ appeared frequently in accessions of aromatic and tropical *japonica*, which exhibits an intermediate AAC, and is determined by a mutation in exon 6 at position +1,083. This mutation altered a Tyr to Ser in the active site of the enzyme and reduced its specific activity. The results of Tran et al. (2011) showed that the Ex10C/T SNP of *Wx* mainly affected GC. Rice with a C at exon 10 had soft, viscous gels once cooked; however, a sample with a T had short, firm gels.

The synthesis of amylopectin is more complex than that of amylose. The enzymes involved in amylopectin synthesis including soluble starch synthase (SSS), starch branching enzyme (SBE), and starch debranching enzyme (DBE) (Ball and Morell, 2003). SSIII is one of the soluble starch synthases and the *SSIII-1* (*SSIIIb*) gene is mainly expressed in leaves, whereas *SSIII-2* (*SSIIIa*) is preferentially expressed in endosperm and is responsible for the synthesis of long-chain amylopectin (Lloyd et al., 1999). Previous studies have found that the long-chain amylopectin content, with a polymerization degree of more than 30, was reduced by about 60% after silencing *SSIII-2*, and it also had some influence on starch GT (Fujita et al., 2007).

Five putative alleles at the *Wx* loci have been identified and indicated to be responsible for the variation of ECQs. However, the combined genetic effects of different *Wx* alleles on grain ECQs remain unclear. Previous studies have not identified the effects of the interaction of *Wx* and the *SSIII-2* gene on rice ECQs. To breed rice cultivars with superior and novel grain qualities to meet the demands of consumers, it is necessary to clarify the correlation between the different *Wx* allele polymorphism and the grain qualities of cultivars, and analyze the interaction of *Wx* and *SSIII-2* on rice quality. In this study, a F_4_ population was derived from a combination of CG133R, a restorer with a high AAC, and glutinous rice (*javanica* 22) as research materials. The effect of different haplotypes of *Wx* and their interaction with *SSIII-2* on ECQs were investigated. This results of this study will be useful for the molecular breeding of rice quality.

## Materials and methods

### Materials

In this study, a hybrid combination of CG133R (restorer, AAC ≈ 28%) and Javanica22 (a natural mutant from *javanica* rice variety Xiangdali, AAC ≈ 0.7%) was established. The alleles of the two parents differed in terms of *AGPlar, AGPsma, Wx*, and the *SSIII-2* gene. In the third generation, 68 plants that differed in *Wx* and *SSIII-2* loci were selected as research materials. In the fourth generation, a line of 120 plants was selected as the research material. These plants only had polymorphism on the *Wx* locus and came from the F_3_ generation. These plants were planted in an experimental agricultural field at the Southwest University of Science and Technology and were maintained regularly. At maturity, the seeds from each plant were harvested for the investigation of physical and chemical indicators and RVA profile characteristics.

### Methods

#### DNA extraction

About 100 mg rice leaf tissues were collected from the main stem of each individual plant at the tillering stage and were ground using a Fastprep Sample Rapid Crushing System (MP Biomedicals, Santa Ana, CA, USA) for DNA extraction as described previously (Murray and Thompson, 1980).

#### DNA amplification and restriction digestion

PCR-AccI and WXE10, which were developed by Cai et al. (2002) and Tran et al. (2011), were used to detect the +1 base type of the first intron and the tenth exon C/T polymorphism of *Wx*, respectively. To genotype the SNP on exon 6 of *Wx*, primers of Exon6 were designed exactly as described by Dobo et al. (2010). SSIII-2 M1 was designed to detect A/G polymorphism according to Tian et al. (2010). All the primers were synthesized by Sangon Biotech (Shanghai, China).

The PCR consisted of a 15 μL reaction mixture with 2 μL of template DNA, 7.5 μL of 2 × Reaction Mix, 1.5 μL of 1 μmol L^−1^ primer, 0.2 μL of 2.5 U μL^−1^ Golden DNA Polymerase and 3.8 μL of ddH_2_O. The PCR reaction was performed using a BIO RAD DNA Engine Dyad Peltier Thermal Cycler (BIO RAD, Hercules, CA, USA).

The PCR-ACCI and WXE10 amplification products were digested with restriction endonuclease ACCI and ApaI, respectively, in a 15 μL reaction mixture containing 10 μL of amplification product, 1.5 μL of 10 × buffer, 0.5 μL of restriction endonuclease, and 3 μL of ddH_2_O. The digest reaction was performed at 37°C for 3 h.

The products of WXE10, EXon6, PCR-AccI, and SSIII-2 M1 were detected using 2.5% agarose gel in 0.5 × Tris-Borate EDTA (TBE) buffer. The gel was run at 150 V for 40 min and stained with GreenView. The size of the amplification products was estimated using a 100 bp DNA ladder.

#### Determination of physical and chemical indexes of starch

AAC was assayed by the colorimetric method with iodine-potassium iodide. GC was measured according to Chinese national standard GB/T 17891–1999. In the third generation, GT was assayed by the alkaline spreading value (ASV) method according to GB/T 17891–1999. In the fourth generation, GT was measured with differential scanning calorimetry (DSC) as described by Yan et al. (2010). Each sample was tested three times.

#### RVA profile characteristics measurement

RVA profile characteristics were measured using a rapid RVA (Model No. RVA4500, NewPortSci. Co., Warriewood, Australia), according to the American Association of Cereal Chemists standard method AACC61-02. Rice starch viscosity characteristics included the following original parameters: peak viscosity (PKV), hot paste viscosity (HPV), and cool paste viscosity (CPV), and three secondary parameters: breakdown (BDV), setback (SBV), and consistency (CSV). In addition, pasting temperature (PaT) and pasting time (PeT) were also recorded. Each measurement was repeated twice.

#### Statistical analysis

Data were classified according to the genotyping results. An analysis of variance (ANOVA) was performed using the Data Processing System version 9.50 (DPS, http://www.chinadps.net). Multiple comparisons of the Duncan method were performed using the Statistical Product and Service Solutions (SPSS) software version 19.

## Results

### Effects of *SSIII-2* under different *Wx* background

There was no obvious difference in the tested characteristic values between different *SSIII-2* alleles under the same background of *Wx*^*a*^ (SNP of *Wx* loci with G) (data not shown). However, significant differences under the background of *Wx*^*b*^ (SNP of *Wx* loci with T) were detected (Table 1). The results of a multiple comparison showed that the ASV, PKV, BDV, and PaT varied significantly between different polymorphisms of the *SSIII-2* alleles. The ASV of SSIII-2-II type (same as CG133R) increased by 144.54%, and its PKV, BDV, and PaT decreased by 14.58, 36, and 7.23%, respectively, compared with the SSIII-2-I type (same as Javanica22). The effects of *SSIII-2* alleles on rice quality varied under the different backgrounds of *Wx* alleles, which implied an interaction between *Wx* and *SSIII-2*.

**Table 1:** Comparison of effects of different *SSIII-2* alleles under background of *Wx*^*b*^.

**Genotype**	**ASV**		**PKV/RVU**		**BDV/RVU**		**PaT/**^**°**^**C**	
	**Mean ± SD**	**Range**	**Mean ± SD**	**Range**	**Mean ± SD**	**Range**	**Mean ± SD**	**Range**
I	2.29 ± 0.41	2.00~2.58	299.11 ± 4.45^**^	293.79~304.42	113.23 ± 0.21^**^	113.08~113.38	78.28 ± 0.60^**^	77.85~78.70
II	5.60 ± 0.35^**^	5.20~5.85	256.46 ± 8.34	248.56~265.33	73.18 ± 7.05	66.51~80.56	72.36 ± 0.81	71.45~73.01

** *Significant at 1% level*.

### Interaction effect of *Wx* and *SSIII-2*

The differences in AAC and ASV between the partial combinations of different *Wx* alleles and *SSIII-2* (Table 2) were both extremely significant. The AAC of the genotype of *Wx*^*b*^-I and *Wx*^*b*^-II was significantly lower than that of *Wx*^*a*^-I and *Wx*^*a*^-II. However, there was no significant difference in the AAC between the genotypes of *Wx*^*a*^-I and *Wx*^*a*^-II. Moreover, the AAC of the *Wx*^*b*^-I genotype was similar to that of *Wx*^*b*^-II. The ASV of *Wx*^*b*^-I was significantly lower than that of *Wx*^*b*^-II, while there was no significant difference in the ASV of *Wx*^*a*^-I and *Wx*^*a*^-II.

**Table 2:** Comparison of physicochemical indexes among different combinations of *Wx* and *SSIII-2* alleles.

**Genotypes**		**AAC/%**	**GC/mm**	**ASV**
***Wx***	***SSIII-2***			
*Wx^*a*^*	I	30.99 ± 3.41^A^	100.37 ± 19.60	3.93 ± 1.86^AB^
	II	29.76 ± 2.92^A^	96.32 ± 23.31	3.99 ± 1.44^AB^
*Wx^*b*^*	I	15.45 ± 3.18^B^	122.60 ± 25.88	2.29 ± 0.41^B^
	II	17.19 ± 1.34^B^	106.46 ± 12.06	5.60 ± 0.35^A^

*Lowercase and capital letters denote significant at 0.05 and 0.01 levels, respectively*.

The RVA profile characteristics of rice for different combinations of the *Wx* and *SSIII-2* alleles were analyzed by multiple comparisons (Table 3). We found significant differences in the PKV, BDV, and PaT among the combinations. The PKV and PaT of *Wx*^*b*^-II were significantly lower than the corresponding values of *Wx*^*b*^-I, *Wx*^*a*^-I, and *Wx*^*a*^-II. The BDV of *Wx*^*b*^-II was also significantly lower than that of *Wx*^*b*^-I, but there was no significant difference in the BDV of *Wx*^*a*^-I and *Wx*^*a*^-II.

**Table 3:** Comparison of RVA profile characteristics among different combinations of *Wx* and *SSIII-2*.

**Genotypes**		**PKV/RVU**	**BDV/RVU**	**PaT/°C**
***Wx***	***SSIII-2***			
*Wx^*a*^*	I	285.49 ± 29.91^A^	87.67 ± 18.05^AB^	78.62 ± 3.47^A^
	II	295.91 ± 27.47^A^	96.52 ± 28.59^AB^	79.04 ± 3.86^A^
*Wx^*b*^*	I	299.11 ± 4.45^A^	113.23 ± 2.12^A^	78.28 ± 0.60^A^
	II	256.46 ± 8.34^B^	73.18 ± 7.05^B^	72.36 ± 0.81^B^

*Lowercase and capital letters denote significant at 0.05 and 0.01 levels, respectively*.

### Combined effects of *Wx* (In1G/T) and *Wx* (Ex6A/C)

As shown in Table 4, five haplotypes of *Wx* were identified by the different SNPs of *Wx* (In1G/T) and *Wx* (Ex6A/C): GG-AA, GG-CC, GT-AA, GT-CC, and TT-AA. There was no significant difference in their GC and GT. However, there was a significant difference in their AAC. The mean AAC was only 8.08% (range of 7.15–9.01%) in the rice line with haplotype TT-AA, whereas the mean AAC in the other four haplotypes was in the range of 26.60–30.19%.

**Table 4:** ECQs for different haplotypes of In1G/T and Ex6A/C polymorphisms of *Wx*.

**Loci of SNP**		**AAC (%)**		**GC (mm)**		**GT (**^**°**^**C)**	
**In1**	**Ex6**	**Mean ± SD**	**Range**	**Mean ± SD**	**Range**	**Mean ± SD**	**Range**
GG	AA	30.19 ± 6.54^A^	19.11–39.22	99.32 ± 22.83	53.00–125.50	72.15 ± 7.23	60.29–79.63
GG	CC	29.78 ± 6.02^A^	24.19–35.70	103.96 ± 31.50	67.50–140.30	69.54 ± 2.80	66.31–71.21
GT	AA	27.75 ± 8.87^A^	15.99–35.93	100.44 ± 17.02	72.25–119.90	69.81 ± 5.98	63.99–77.23
GT	CC	26.60 ± 7.63^A^	15.29–31.98	103.50 ± 13.84	92.20–123.25	70.69 ± 3.88	68.28–75.16
TT	AA	8.08 ± 1.32^B^	7.15–9.01	103.65 ± 12.94	94.50–112.80	74.28 ± 3.72	6953–78.03

*Lowercase and capital letters denote significant at 0.05 and 0.01 levels, respectively*.

**Figure 1:**
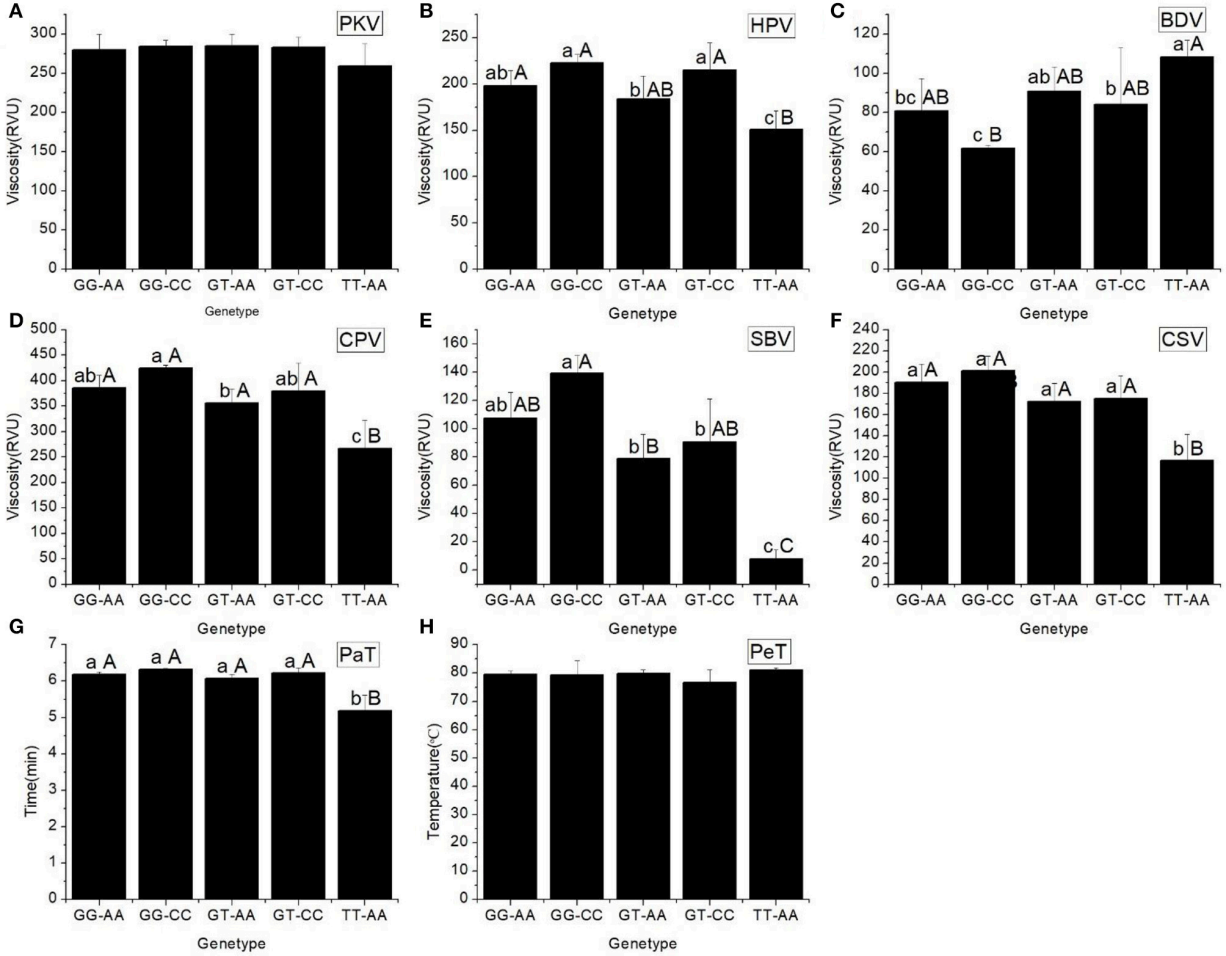
RVA profile characteristics for different haplotypes of In1G/T and Ex6A/C polymorphisms of *Wx*. **(A)** Peak viscosity (PKV). **(B)** Hot paste viscosity (HPV). **(C)** Cool paste viscosity (CPV). **(D)** Breakdown valve (BDV). **(E)** Setback valve (SBV). **(F)** Consistency valve (CSV). **(G)** Pasting temperature (PaT). **(H)** Pasting time (PeT). GG-AA, GG-CC, GT-AA, GT-CC and TT-AA represent different haplotypes of In1G/T and Ex6A/C polymorphisms of *Wx*. Lowercase and capital letters above column denote significant at 0.05 and 0.01 levels, respectively.

All the RVA profile characteristics are provided in Figure 1. The HPV, CPV, SBV, SCV, and PaT of haplotype TT-AA were significantly different to those of the other haplotypes. However, the BDV of haplotype GG-CC was significantly less than that of the other haplotypes.

### Combined effects of *Wx* (In1G/T) and *Wx* (Ex10C/T)

The tested materials had six combinations of different SNPs of *Wx* (In1G/T) and *Wx* (Ex10C/T) (Table 5). The results of a multiple comparison showed that AAC and GC varied significantly among the different haplotypes. The haplotype TT-CC had the highest GC and the lowest AAC, and the haplotype GG-TT had the highest AAC and lowest GC.

**Table 5:** Rice ECQs for different haplotypes of In1G/T and Ex10C/T polymorphisms of *Wx*.

**Loci of SNP**		**AAC (%)**		**GC (mm)**		**GT (**^**°**^**C)**	
**In1**	**Ex10**	**Mean ± SD**	**Range**	**Mean ± SD**	**Range**	**Mean ± SD**	**Range**
GG	CC	29.07 ± 5.39^A^	22.27–38.49	108.42 ± 18.24^ab^	82.55–142.60	73.83 ± 4.58	68.84–79.63
GG	TT	30.03 ± 5.65^A^	19.11–37.25	94.21 ± 19.47^b^	59.25–118.55	70.60 ± 5.71	65.67–78.82
TT	CC	5.54 ± 3.21^B^	1.45–9.01	114.08 ± 15.43^a^	94.50–132.00	78.14 ± 1.70	76.94–79.35
TT	TT	8.03 ± 4.15^B^	3.84–13.95	110.22 ± 28.53^a^	66.55–133.55	75.84 ± 2.63	71.84–78.80
GT	CC	24.91 ± 4.89^A^	15.99–29.83	103.27 ± 27.54^ab^	69.75–135.60	73.04 ± 4.92	68.63–79.01
GT	TT	25.76 ± 5.37^A^	18.37–29.30	100.73 ± 18.20^ab^	63.00–125.10	70.56 ± 4.83	63.99–77.23

*Lowercase and capital letters denote significant at 0.05 and 0.01 levels, respectively*.

**Figure 2:**
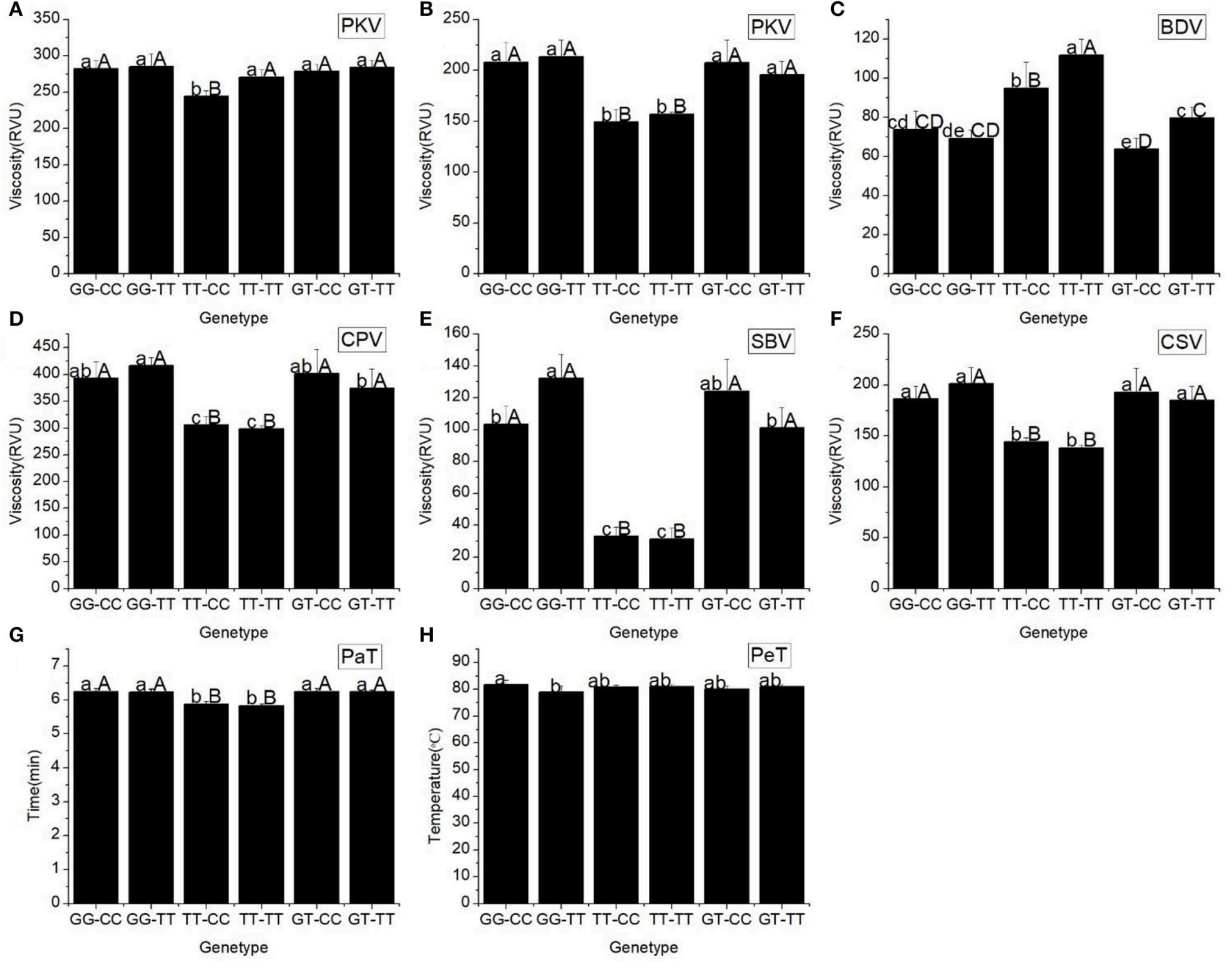
RVA profile characteristics for different haplotypes of In1G/T and Ex10C/T polymorphisms of *Wx*. **(A)** Peak viscosity (PKV). **(B)** Hot paste viscosity (HPV). **(C)** Cool paste viscosity (CPV). **(D)** Breakdown valve (BDV). **(E)** Setback valve (SBV). **(F)** Consistency valve (CSV). **(G)** Pasting temperature (PaT). **(H)** Pasting time (PeT). GG-CC, GG-TT, TT-CC, TT-CC, GT-CC and GT-TT represent different haplotypes of In1G/T and Ex10C/T polymorphisms of *Wx*. Lowercase and capital letters above column denote significant at 0.05 and 0.01 levels, respectively.

Our results showed that the starch pasting properties of the different haplotypes of *Wx* (In1G/T SNP) and *Wx* (Ex10C/T SNP) altered significantly (Figure 2). Three basic parameters of the RVA profile (PKV, HPV, and CPV), two secondary parameters (SBV and CSV), and the PaT of haplotypes TT-CC and TT-TT dramatically decreased compared with the other haplotypes. The BDV of TT-TT was the highest among the different haplotypes. When compared with the other haplotypes, the PeT of haplotypes GG-CC and GG-TT were the highest and lowest, respectively.

### Combined effects of *Wx* (Ex6A/C) and *Wx* (Ex10C/T)

Four haplotypes, AA-CC, AA-TT, CC-CC, and CC-TT, which originated from the combination of *Wx* (Ex6A/C) and *W*x (Ex10C/T) (Table 6), differed from each other in terms of GC among the line we tested. Their GC was significantly different at the 0.01 level. The haplotype AA-CC had the longest gel length (113.58 ± 22.94 mm), whereas the haplotype CC-CC had the shortest gel length (81.83 ± 18.66 mm).

**Table 6:** ECQs for different haplotypes of Ex6A/C and Ex10C/T polymorphisms of *Wx*.

**Loci of SNP**		**AAC (%)**		**GC (mm)**		**GT (**^**°**^**C)**	
**In6**	**Ex10**	**Mean ± SD**	**Range**	**Mean ± SD**	**Range**	**Mean ± SD**	**Range**
AA	CC	21.50 ± 10.40	4.84–31.49	113.58 ± 22.94^A^	72.25–141.00	73.35 ± 4.26	68.84–79.01
AA	TT	26.30 ± 10.01	4.36–37.25	97.72 ± 20.28^AB^	59.25–124.65	69.64 ± 5.38	60.29–78.85
CC	CC	29.38 ± 4.26	23.26–32.85	81.83 ± 18.66^B^	55.05–96.00	72.75 ± 3.90	68.63–75.50
CC	TT	27.73 ± 5.14	15.29–35.70	97.25 ± 22.26^AB^	67.50–116.80	70.65 ± 3.80	66.31–77.33

*Lowercase and capital letters denote significant at 0.05 and 0.01 levels, respectively*.

**Figure 3:**
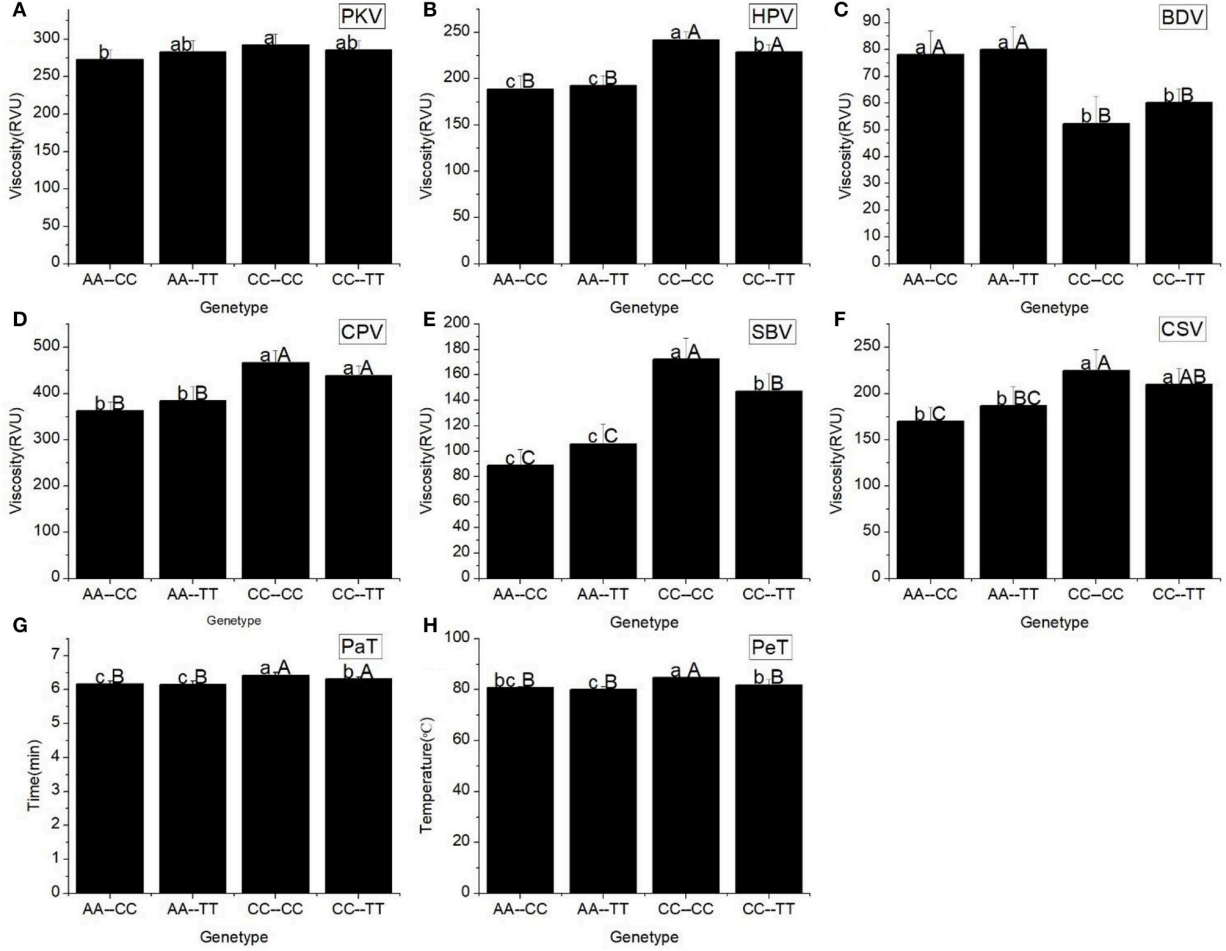
RVA profile characteristics for different haplotypes of Ex6A/C and Ex10C/T polymorphisms of *Wx*. **(A)** Peak viscosity (PKV). **(B)** Hot paste viscosity (HPV). **(C)** Cool paste viscosity (CPV). **(D)** Breakdown valve (BDV). **(E)** Setback valve (SBV). **(F)** Consistency valve (CSV). **(G)** Pasting temperature (PaT). **(H)** Pasting time (PeT). AA-CC, AA-TT, CC-CC and CC-TT represent different haplotypes of Ex6A/C and Ex10C/T polymorphisms of *Wx*. Lowercase and capital letters above column denote significant at 0.05 and 0.01 levels, respectively.

All RVA profile characteristics, except HPV, were significantly different (Figure 3) among the different Haplotypes. The HPV, CPV, SBV, CSV, PaT, and PeT of haplotypes CC-CC and CC-TT were significantly higher than the corresponding values for the other haplotypes. However, there was no difference in these RVA parameters between the haplotypes CC-CC and CC-TT, as well as between the haplotypes AA-CC and AA-TT.

### Combined effects of *Wx* (In1G/T), *Wx* (Ex6A/C) and *Wx* (Ex10C/T)

According to the results of a multiple comparison (Table 7), ten haplotypes of *Wx* were identified by their different SNPs. The results indicated that a significant difference in AAC and GC existed among the different haplotypes. The TT-AA-CC and TT-AA-TT haplotypes had a low AAC (6.09 and 8.03%, respectively) and a soft GC (114.08 and 111.87 mm, respectively). The GC of haplotypes GG-AA-CC and GG-CC-CC were the hardest and softest, respectively.

**Table 7:** ECQs for different haplotypes of In1G/T, Ex6A/C and Ex10C/T polymorphisms of *Wx*.

**Loci of SNP**			**AAC (%)**		**GC (mm)**		**GT (**^**°**^**C)**	
**In1**	**In6**	**Ex10**	**Mean ± SD**	**Range**	**Mean ± SD**	**Range**	**Mean ± SD**	**Range**
GG	AA	CC	28.18 ± 5.86^A^	22.27–38.49	120.52 ± 13.47^aA^	97.15–130.60	74.38 ± 5.36	68.84–79.63
GG	AA	TT	30.44 ± 5.51^A^	19.11–39.22	93.74 ± 12.84^bcAB^	78.30–109.10	70.29 ± 5.14	64.56–79.83
GG	CC	CC	32.21 ± 0.91^A^	31.57–32.85	74.02 ± 8.83^cB^	65.05–83.00	72.75 ± 3.90	69.99–75.00
GG	CC	TT	28.87 ± 4.52^A^	25.00–35.70	98.82 ± 12.94^abAB^	88.80–116.80	70.53 ± 3.88	66.31–77.33
TT	AA	CC	6.90 ± 2.21^B^	4.84–9.01	114.08 ± 15.43^abA^	94.50–132.00	78.14 ± 1.70	76.94–79.35
TT	AA	TT	8.03 ± 3.15^B^	3.84–12.21	111.87 ± 16.14^abA^	95.50–133.55	75.84 ± 1.63	73.44–78.02
GT	AA	CC	24.37 ± 4.74^A^	15.99–29.42	109.04 ± 26.92^abA^	72.25–135.60	74.51 ± 4.83	69.40–79.01
GT	AA	TT	25.43 ± 7.59^A^	18.37–32.09	104.25 ± 14.38^abAB^	78.50–119.90	69.61 ± 5.79	63.99–77.23
GT	CC	CC	26.54 ± 3.65^A^	23.26–29.83	89.63 ± 9.02^bcAB^	83.25–96.00	68.63 ± 1.25	66.90–70.33
GT	CC	TT	26.41 ± 5.91^A^	15.29–31.98	94.04 ± 14.52^bcAB^	74.35–107.05	71.96 ± 3.13	68.28–75.16

*Lowercase and capital letters denote significant at 0.05 and 0.01 levels, respectively*.

**Figure 4:**
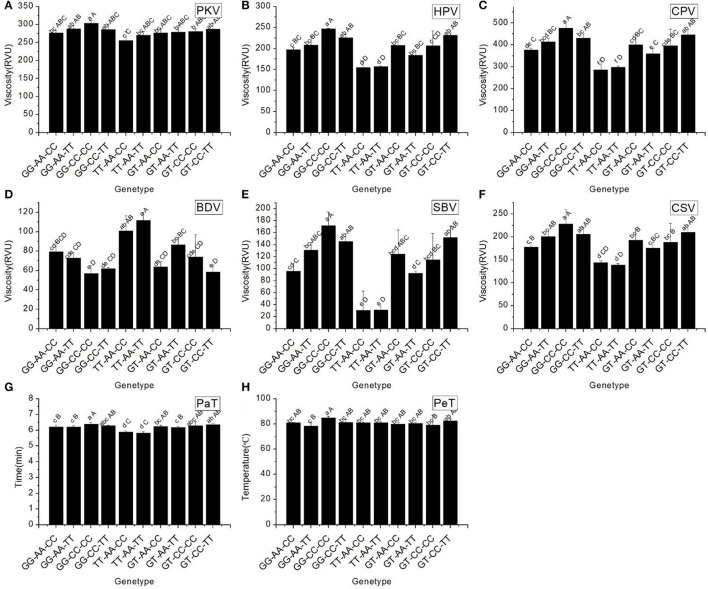
RVA profile characteristics for different haplotypes of In1G/T, Ex6A/C and Ex10C/T polymorphisms of *Wx*. **(A)** Peak viscosity (PKV). **(B)** Hot paste viscosity (HPV). **(C) C**ool paste viscosity (CPV). **(D)** Breakdown valve (BDV). **(E)** Setback valve (SBV). **(F)** Consistency valve (CSV). **(G)** Pasting temperature (PaT). **(H)** Pasting time (PeT). GG-AA-CC, GG-AA-TT, GG-CC-CC, GG-CC-TT, TT-AA-CC, TT-AA-TT, GT-AA-TT, GT-AA-TT, GT-CC-CC and GT-CC-TT represent different haplotypes of In1G/T, Ex6A/C and Ex10C/T polymorphisms of *Wx*. Lowercase and capital letters above column denote significant at 0.05 and 0.01 levels, respectively.

RVA profile characteristics varied significantly among the different haplotypes (Figure 4). It should be noted that the maximum values of all RVA profile characteristics appeared in haplotype GG-CC-CC, except for BDV. The minimum values of two original parameters (PKV and HPV) and the secondary parameters (CPV and SBV) were found in haplotype TT-AA-CC. These results indicated that different combinations of the SNPs of *Wx* had a remarkable influence on the profile of RVA parameters.

## Discussion

As one of the most important objectives in rice breeding, ECQs has received increasing attention in recent years. However, due to the complexity in the quality inheritance, as well as the lack of effective measurements for breeding high quality rice, many high-yielding varieties are of poor quality. An understanding of the molecular mechanism involved in the formation of rice quality is essential for rice quality improvement. Generally, the amylopectin chain length and amylose molecular size have a synergistic effect on the quality of starch. Thus, a clarification of the effects of starch synthesis-related genes on grain quality is very important for quality breeding.

The results of this investigation indicated that *SSIII-2* significantly affected the GT, PKV, BDV, and PaT of rice under the background of *Wx*^*b*^. The *SSIII-2* gene contains 15 introns and 16 exons, and its third exon has repeated sequence polymorphism (Dian et al., 2005). Li et al. (2000) found that *SSIII-2* was responsible for the synthesis of long-chain amylopectin in endosperm. Liu (2012) also found that *SSIII-2* had a significant effect on rice GT by studying its near isogenic lines (NILs).

The biosynthesis of rice starch requires the association of multiple genes. For example, *SSIII-2* not only participates in the synthesis of long-chain amylopectin, but also regulates the activity and expression level of SSS isozyme. Silencing *SSIII-2* has been shown to result in an increase in SSI activity in rice (Fujita et al., 2007). Wu et al. (2006) found that *SSI, SSII-3, SSIII-1*, and *SBE* had no significant effect on rice quality under the background of *Wx*^*a*^, while there were obvious effects when *Wx*^*a*^ was replaced by the recessive gene *wx*. Yan et al. (2006) found that the genetic effects of *SBEI* and *SBEIII* under different backgrounds (*Wx*^*a*^ and *Wx*^*b*^) varied. The genetic effects of *SBEI* and *SBEIII* might be masked by *Wx*. Although *SSIII-2* had no significant effect on rice quality under the background of *Wx*^*a*^, it had very significant effects on GT, PKV, BDV, and PaT under the background of *Wx*^*b*^. The expression level of *Wx*^*b*^ genotype was significantly lower than that of *Wx*^*a*^ genotype due to the first intron mutation of *Wx*^*a*^. This implies that the effect of *SSIII-2* may be masked by the massive synthesis of GBSS under the *Wx*^*a*^ genotype, which is a dominant controlling factor of rice quality and has a dominant epistasis effect on *SSIII-2*.

It is generally accepted that *Wx* is the major gene that controls rice AAC, and its three SNPs and one CT microsatellite repeat have been found in non-glutinous rice. Ayres et al. (1997) found that an SNP of In1G/T explained almost 80% of the variation in amylose content of their test materials. The genotype In1TT produced a low amylose content, while the AAC of genotype In1GG was significantly higher than that of genotype In1TT. Larkin and Park (2003) conducted a sequence analysis and found an A/C polymorphism in exon 6 and a C/T polymorphism in exon 10, which resulted in a non-conservative residue substitution. Plants with an A-C mutation in exon 6 led to an alteration of amino acid from Tyr to Ser and an intermediate AAC. The SNP Ex10TT was present only in rice with a high AAC. Tran et al. (2011) found that Ex10C/T could explain a significant component of gel consistency. In our study, a combination of In1G/T and Ex6A/C had a significant impact on AAC and no effect on GC. The combination of In1G/T and Ex10C/T had a significant effect on both AAC and GC. However, the combination of Ex6A/C and Ex10C/T had no effect on AAC but did have an effect on GC. This suggested that AAC was mainly controlled by In1G/T and rice GC was regulated by Ex10C/T.

The combined effect of different SNPs plays an important role in determining rice grain qualities. Our study showed that In1T-Ex6A-Ex10C (mean AAC = 6.90%) and In1T-Ex6A-Ex10T (mean AAC = 8.03%) were classified as the low AAC type, while the other haplotypes belonged to the high AAC type (>23%). The combined effects of three SNPs had a significant influence on all ECQs and RVA profile parameters except for GT. Dobo et al. (2010) identified three SNPs of *Wx* and measured its AAC in the US and European rice germplasm. Their results showed that a combination of these three SNPs accounted for 89.2% of the variation in the AAC of 85 US germplasm and 93.8% of the variation among 279 European rice varieties. The haplotypes In1T-Ex6A-Ex10C and In1T-Ex6C-Ex10C were found in low AAC varieties, and all varieties with intermediate levels of AAC had the In1G-Ex6C-Ex10C allele. High levels of AAC varieties had either the In1G-Ex6A-Ex10T or In1G-Ex6A-Ex10C allele. It has been reported that AAC and GC are mainly controlled by *Wx*, which is a minor gene affecting GT. SSIIa (SSII-3, ALK) was a dominant factor for controlling the GT (Gao et al., 2003; Umemoto and Aoki, 2005). In the present study, all combinations had no effect on GT. Thus, the same conclusion was reached, i.e., the different combination of the three SNPs of *Wx* had no significant influence on GT.

A previous study indicated that RVA profile parameters were closely related to AAC and GC (Umemoto et al., 2002) as well as *Wx* genes. Zhang et al. (2012) found a significant effect on starch paste viscosity using four NILs with different *Wx* alleles as research materials. Our results clearly demonstrated that the RVA profile parameters of the different combinations of In1G/T and Ex6A/C were significantly different, except for PKV and PeT. This result shows that combined effects of different SNPs of *Wx* are important for rice quality breeding. There is a need to further explore the interaction of different genes that regulate ECQs to enhance the efficiency of quality breeding.

## Author contributions

BY and SX performed the genetic studies. LX and HY carried out the measure of physicochemical indicators and RVA profile characteristics. XX designed the overall project. BY analyzed the data and wrote the manuscript. All the authors read and approved the final manuscript.

### Conflict of interest statement

The authors declare that the research was conducted in the absence of any commercial or financial relationships that could be construed as a potential conflict of interest.
